# The Odour of Sex: Sex-Related Differences in Volatile Compound Composition among Barn Swallow Eggs Carrying Embryos of Either Sex

**DOI:** 10.1371/journal.pone.0165055

**Published:** 2016-11-16

**Authors:** Alessandra Costanzo, Sara Panseri, Annamaria Giorgi, Andrea Romano, Manuela Caprioli, Nicola Saino

**Affiliations:** 1 Department of Biosciences, University of Milan, Milan, Italy; 2 Department of Veterinary Science and Public Health, University of Milan, Milan, Italy; 3 Centre for Applied Studies in the Sustainable Management and Protection of the Mountain Environment, Ge.S.Di.Mont., University of Milan, Edolo, Brescia, Italy; Estacion Experimental de Zonas Aridas, SPAIN

## Abstract

Avian communication has been traditionally believed to be mainly mediated by visual and auditory channels. However, an increasing number of studies are disclosing the role of olfaction in the interaction of birds with their social environment and with other species, as well as in other behaviors such as nest recognition, food location and navigation. Olfaction has also been suggested to play a role in parent-offspring communication not only in the post- but also in the pre-hatching period. Volatile compounds produced during embryogenesis and passively released through the eggshell pores may indeed represent the only cue at parents’ disposal to assess offspring quality, including the sex composition of their clutch before hatching. In turn, sex identification before hatching may mediate adaptive strategies of allocation to either sex. In the present study, we analyzed odour composition of barn swallow eggs incubated in their nest in order to identify any sex-related differences in volatile compounds emitted. For the first time in any bird species, we also investigated whether odour composition is associated with relatedness. The evidence of differences in odour composition among eggs containing embryos of either sex indicates that parents have a cue to identify their brood sex composition even before hatching which can be used to modulate their behavior accordingly. Moreover, odour similarity within nests may represent the prerequisite for kin recognition in this species.

## Introduction

Acoustic and visual channels are known to play a major role in avian communication, as witnessed by the independent evolution of elaborated songs and bright colours in many bird phyla [1–3]. Following the pioneering work of Bang [4] and Wenzel [5], increasing evidence has been gathered that olfaction also plays an important, though largely neglected, role in bird communication. Birds have been historically believed to be anosomic or microsmatic (i.e. having no or little sense of smell) [6], but anatomical evidence strongly supports their functional olfaction [7,8]. Empirical studies of the role of olfaction in birds have initially been focused on species with large olfactory bulbs [9,10], but the interest has then been expanded to species with a smaller ratio between the size of the olfactory bulbs and the rest of the brain, such as passerines [11].

Odours are relevant to the way in which birds interact with their social environment and with other species, by mediating individual and kin recognition [12,13] and interactions with their brood parasites [14]. In addition, olfaction can mediate major behavioural functions such as navigation [15], location of food [16] and identification of the own nest [17].

Discrimination between age classes and sex of conspecifics has also been shown to occur via olfactory cues in birds [18]. The main odour source associated with sex recognition is the uropygial gland secretion, which is composed of a mixture of volatile and non-volatile monoester and diester waxes, tryglicerides, fatty acids and hydrocarbons, that are rubbed on body feathers during the preening behaviour [19]. In both the dark-eyed junko *Junco hyemalis* [20] and the spotless starling *Sturnus unicolor* [18], for example, individuals of either sex do not differ in odour composition of the uropygial secretion but in the relative concentration of the individual compounds, suggesting a potential role of odour in sexual selection [21,22].

Increasing evidence on olfactory discrimination ability of birds discloses the possibility that odour also plays a role in parent-offspring communication, including interactions in both the pre- and the post- hatching periods [14,23–25]. For example, odour may represent one of the very few cues at parents’ disposal in order to identify offspring sex before hatching [26]. Offspring of either sex can differ in their need of care and parents should modulate their investment towards offspring of the sex that will guarantee the largest fitness rewards [27–30]. Differential allocation of parental investment to offspring of either sex requires that offspring sex can be identified since very early life stages, especially in species with a short period of parental care, such as many small passerine birds. Differences in phenotype between male and female offspring have been found for body size [31–33], plumage colouration [34,35], begging vocalizations [36,37] and mouth conspicuousness [38]. However, all these sex-related differences always emerge during offspring rearing, thus reducing the scope for an early identification of the progeny sex.

Because bird eggshell porosity allows the passive diffusion of metabolic gases (such as carbon dioxide, oxygen and water vapour) [39,40] and odorous volatile compounds produced during the development of the embryo [41], sex identification before hatching could be mediated by volatile gas emissions of the eggs. In an extensive literature search, however, we could find only a single study that investigated variation in egg odour, as determined by a mixture of volatile compounds, according to the sex of the embryo. In a study conducted on a captive population of japanese quail (*Coturnix japonica*), Webster and co-workers [26] reported changes in egg volatile compound composition along the incubation period, as well as odour differences between fertile and infertile eggs and between egg carrying a male as compared to a female embryo.

The present study thus aimed at quantifying odour composition of barn swallow (*Hirundo rustica*) eggs incubated in their nest in order to identify any sex-related differences in the volatile compounds released. Moreover, we investigated for the first time in any species whether odour patterns were associated with the nest of origin, potentially reflecting the effect of genetic or micro-habitat effects on odour composition.

## Methods

### Model organism

The barn swallow is a colonial, socially monogamous passerine bird with Holarctic distribution [42]. Breeding occurs between April and August. During the breeding season, each pair produce 1–3 clutches of 2–7 eggs. One egg is laid per day and incubation, by the female only, starts on the penultimate day of laying of the last egg, and lasts 14 days [43,44].

### Field procedures

Nests were visited every third day to ascertain date of clutch completion. To our aim, five entire clutches including a total of 24 eggs were collected on day 10–11 of incubation. Only nests with at least four eggs were considered, in order to increase the chances that embryos of both sex were represented in each clutch.

Since no study has ever investigated egg odour in the wild, the possibility existed that environmental substances deposited on the eggshell confounded sex-related variation in odour composition while enhancing among-nest variation. In order to test for this possibility, we designed an experiment whereby five eggs were collected from another nest and eggshells were divided longitudinally into two halves. One of the two halves was then sealed inside a small plastic bag (Balmar 2000) and kept in the lab while the other half was placed in a nest as close as possible (less than 3 m) to the nest under scrutiny, on the day following that of clutch completion in the focal nest. The control eggshells and the focal eggs were then collected and analysed at the same time. For ethical and conservation reasons, we kept the number of clutches due to be collected at a minimum.

The eggs and the control eggshells were removed from the nests using powder-free nitrile gloves in order to prevent possible contamination, and immediately placed in small plastic bags that were stored in a refrigerated bag until the eggs were analysed, within three hours of collection. The analyses were not confounded by volatile compounds released by the plastic bags, because these did not match with the volatiles released by the eggs. In fact, Headspace Solid-Phase Microextraction (HS-SPME) and Gas Chromatography-Mass Spectrometry (GC/MS) analyses (see below) of the empty plastic bags that were used to store the eggs lead to the detection of just seven volatile hydrocarbons (*o*, *m* and *p*-kylenes; hexane, styrene, caprolactam, triacetin), which were not detected in the eggs.

Egg mass was measured to an accuracy of ±0.1 mg upon start of the analysis of volatile compounds. After the analyses of volatiles were completed, the eggs were dissected to sex the embryos by molecular tools [45].

### Analyses of egg volatile compounds

All analyses were performed according to Manzo and co-workers [46]. For Headspace Solid-Phase Microextraction (HS-SPME), the eggs were placed in a 20 mL headspace glass vial fitted with a cap equipped with a silicone/polytetrafluoroethylene septum (Supelco). At the end of the sample equilibration period (1 h), a conditioned (1.5 h; 280°C) 50/30 μm Divinylbenzene/Carboxen/Polydimethylsiloxane StableFlex™ fiber (Supelco) was exposed to the headspace of the sample for extraction (3 h), using a CombiPAL system injector autosampler (CTC Analytics). An extraction temperature of 20°C was selected in order to prevent alterations of the sample (oxidation of some compounds, particularly aldehydes [47]). The vials were placed on a heater plate (CTC Analytics) to maintain constant temperature (20°C) during analysis. Injections were performed in splitless mode (5 min) for solid-phase microextraction (SPME). All analyses were performed using a Trace GC Ultra (Thermo-Fisher Scientific) Gas Chromatograph coupled with a quadrupole Mass Spectrometer (GC/MS) Trace DSQ (Thermo-Fisher Scientific) and equipped with an Rtx-Wax column (30 m; 0.25 mm i.d.; 0.25 μm film thickness, Restek). The oven temperature program was: from 35°C, hold 8 min, to 60°C at 4°C/min, then from 60°C to 160°C at 6°C/min and finally from 160°C to 200°C at 20°C /min. Carry over and peaks originating from the fiber were regularly assessed by running blank samples. After each analysis, fibers were immediately thermally desorbed in the GC injector for 5 min at 250°C to prevent contamination. The transfer line to the mass spectrometer was maintained at 230°C, and the ion source temperature was set at 250°C. The mass spectra were obtained by using a mass selective detector with the electronic impact at 70 eV, a multiplier voltage of 1456 V, and by collecting the data at a rate of 1 scan·s^−1^ over the m/z range of 30–350. The carrier gas was helium at a constant flow of 1 mL·min^−1^. An *n-alkane* mixture (C8–C22, R 8769, Sigma) was run under the same chromatographic conditions as the samples to calculate the Kovats retention indices of the detected compounds. The compounds were identified by comparing retention times of the chromatographic peaks with those of authentic compounds analysed under the same conditions, when available, or by comparing the Kovats retention indices with literature data. The identification of MS fragmentation patterns was performed either by comparison with those of pure compounds or using the NIST MS spectral data base. Quantification of volatile compounds from eggs headspaces samples was carried out by peak area normalization (expressed in %). The analyses were done in duplicate.

### Statistical analyses

Because the concentration of some of the compounds in some samples was below detection limit, we did not include in the statistical analyses those compounds for which, in more than 30% of the samples, the concentration was undetectable for either sex. Fourteen out of the 45 compounds were thus excluded from the analyses (2,3-hexandione, 5-methyl-2-hexanone, decane, non-2-enal, propan-2-ol, ethanol, 2-methylpropan-1-ol, pent-1-en-3-ol, hexan-2-ol, propanoic acid, 1-methyl-4-prop-1-en-2-ylcyclohexene, 2,2,4-trimethyl-3-oxabicyclo[2.2.2]octane, 4-methyl-1-propan-2-ylcyclohex-3-en-1-ol, 1-methyl-4-prop-1-en-2-ylcyclohexan-1-ol). Furthermore, when the estimated concentration of any specific compound in a sample was identified as an outlier, the sample was excluded from all the analyses of that compound.

To test for sex differences in the composition of the volatile compounds we performed linear mixed models on individual compounds separately, where the effect of sex (factor. male: 1; female: 2) and mass (covariate) of the embryo were included as fixed effects and nest identity was included as a random effect. To test if nest identity significantly contributed to the variation in concentration of individual compounds we ran a likelihood ratio test comparing the log-likelihood values of the models including or, respectively, excluding the effect of nest identity. The models were then simplified by removing embryo mass when its effect was statistically non-significant. Comparisons between eggshell halves that were either kept in the lab or in a nest close to the focal nests in order to test for accumulation of environmental compounds were done by t-tests for paired data.

To reduce the risk of incurring in Type I statistical errors due to multiple testing we used the false discovery rate approach [48]. We will therefore qualify as statistically significant only those tests that were such after false discovery rate correction. However, we emphasize that due to ethical and conservation reasons we kept our destructive sample of eggs at a minimum. Small sample size and large reduction of the statistical power of the tests following false discovery rate adjustment of significant values could thus considerably increase the risk of Type II statistical errors. As recommended by Garamszegi [49], and Nakagawa and Cuthill [50], in interpreting the results of the statistical tests of sex-dependent differences in odour composition we also relied on the inspection of effect sizes. For individual analysis from linear mixed models, according to Nakagawa and Cuthill [50], these were computed as $d=\frac{t_{MEM}\left[1+\left(\frac{n_{1}}{n_{0}}\right)R\right]\sqrt{1-R}\left(n_{01}+n_{02}\right)}{\sqrt{n_{01}+n_{02}}\sqrt{n_{0}-k}}$, where t_MEM_ is t value from mixed-effects model, n_01_ and n_02_ are the numbers of observations in each treatment, n_0_ is the total number of observations, n_i_ is the number of individuals (or groups), k is the number of parameters (including the intercept) and R is the repeatability or intraclass correlation coefficient. These values has been subsequently converted into Pearson correlation coefficient r according to Cohen [53], as $r=\frac{d}{\sqrt{d^{2}+4}}$. Effect sizes were also computed for the paired t-tests comparing the concentration of volatile compounds between control eggshells that were kept in the lab or, respectively, in an empty nest nearby our focal nests. Here, effect sizes were computed as $r=\sqrt{\frac{t^{2}}{t^{2}+df}}$ following Rosenberg et al. [51]. Mean effect sizes (*Z*_*r*_) and their 95% confidence intervals were computed according to Viechtbauer [52], after the conversion of the r values in *Z*_*r*_ by using the Fisher Z-transformation. Following Cohen [53], effect sizes (r) larger than 0.5 were considered as diagnostic of “large” effect of sex, those ranging between 0.3–0.5 as “intermediate” while those < 0.3 as “small”. All the analyses were carried out in SPSS statistics (version 21.0) and the degree of freedom were computed by using the Satterthwaite's approximation. The false discovery rate and the mean effect sizes (*Z*_*r*_) were calculated in R (version 3.1.0).

### Ethics statement

The study was authorized by Regione Lombardia (Decreto n° 2959, issued on April 5, 2012). The study was conducted in private lands, and land owners gave us the permission to access their farms. The eggs were collected and immediately placed in a plastic bag in a cool bag while in the field. No approval by Animal Ethics Committee was required for the present experimental protocol. No specific review or approval was required for obtaining the permit of collecting the eggs.

## Results

**Fig 1:**
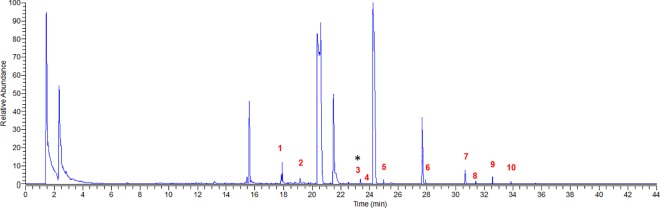
Representative chromatogram of volatile compounds emitted by the eggs. Numbers above peaks indicate volatile compounds significantly different between sexes before false discovery rate adjustment (1: pentadecane; 2: tetradecane; 3: (1R,3R,4S)-2,2,4-trimethyl bicyclo[2.2.1]heptan-3-ol; 4: formamide; 5: 5-methyl-3-hexanol; 6: decanal; 7: formic acid; 8: 2-methylpropanoic acid; 9: 5-(dithiolan-3-yl)pentanoic acid; 10: 2-ethylhexanoic acid). Asterisk above peak indicates the volatile compound significantly different between sexes after false discovery rate adjustment.

The sample consisted of a total of 24 eggs, from 5 nests belonging to 3 colonies. Three eggs were infertile, 13 contained a male and 8 a female embryo. Egg mass was significantly larger in eggs containing a female as compared to a male embryo (t = 2.28, df = 17, p = 0.036), while the difference in body mass between embryos of either sex was marginally non-significant (t = 2.10, df = 17, p = 0.051), with male embryos being heavier than females. Two eggs, one for each sex and belonging to two nests, were excluded from all the analyses because their volatile composition was inconsistent with the general pattern of composition of the eggs, perhaps due to the embryo being dead at the time of collection. The 19 remaining eggs included in the analyses were still representative of all the 5 sampled nests. We identified a total of 45 volatile compounds (Fig 1), belonging to 7 classes, including: ketones (5 compounds: propan-2-one; 6-methyl-5-heptan-2-one; 2,3-hexandione; 5-methyl-2-hexanone; 1-phenylethanone); hydrocarbons (3 compounds: decane; pentadecane; tetradecane); terpenes (7 compounds: 1-methyl-4-prop-1-en-2-ylcyclohexene; 2,2,4-trimethyl-3-oxabicyclo[2.2.2]octane; 4,7,7-trimethylbicyclo[2.2.1]heptan-3-one; (4,7,7-trimethyl-3-bicyclo[2.2.1]heptanyl)acetate; 4-methyl-1-propan-2-ylcyclohex-3-en-1-ol; 1-methyl-4-prop-1-en-2-ylcyclohexan-1-ol; (1R,3R,4S)-2,2,4-trimethylbicyclo[2.2.1]heptan-3-ol); ammides (2 compounds: acetamide; formamide); alcohols (12 compounds: propan-2-ol; ethanol; 2-methylpropan-1-ol; pent-1-en-3-ol; methanethiol; hexan-3-ol; 3-methyl-1-butanol; butane-1,3-diol; hexan-2-ol; 6-ethyl-3-octanol; 5-methyl-3-hexanol; phenol); aldehydes (6 compounds: hexanal; non-2-enal; heptanal; octanal; nonanal; decanal); free fatty acids (10 compounds: 2-methylpropanoic acid; acetic acid; formic acid; propanoic acid; 2-methylbutanoic acid; 2,2-dimethylpropanoic acid; butanoic acid; 5-(dithiolan-3-yl) pentanoic acid; 2-ethylhexanoic acid; octanoic acid).

**Fig 2:**
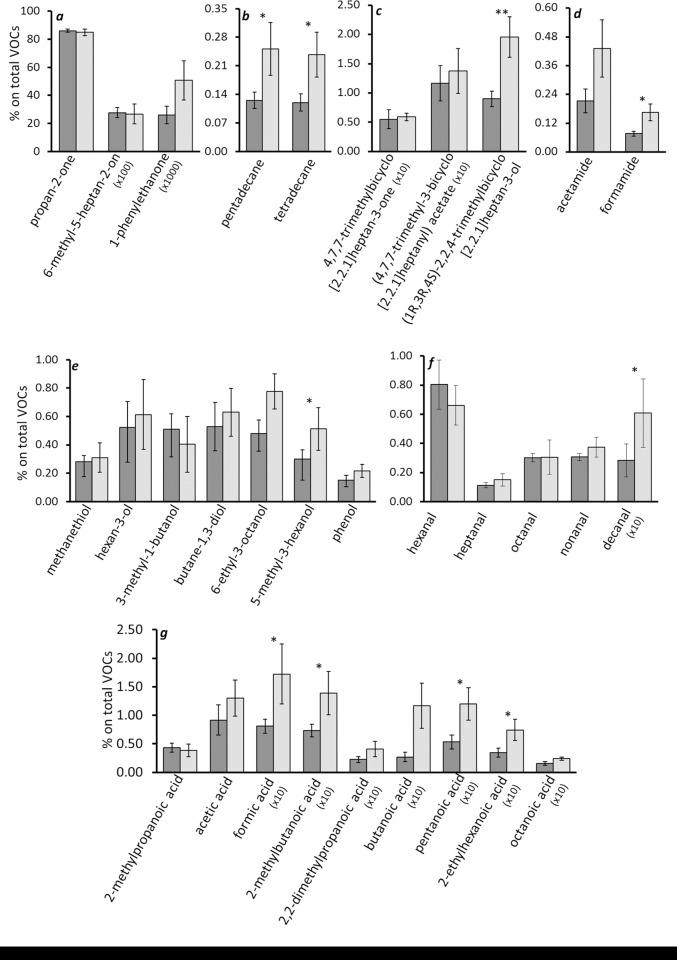
Mean (± SE) percentage on total volatile organic compounds (% on total VOCs) in the two sexes (male: dark grey; female: light grey). Volatile compounds are grouped according to their classes (a. ketones; b. hydrocarbons; c. terpenes; d. ammides; e. alcohols; f. aldehydes; g. free fatty acids). Asterisks indicate significant differences in the concentration of the volatile compounds between sexes. Double asterisks indicate that the volatile compound significantly differed between sexes after false discovery rate adjustment.

For 10 out of the 31 compounds included in the statistical analyses (see Methods), the effect of sex from linear mixed models with nest as a random effect was found to be associated with p < 0.05 to concentration of volatile compounds (Table 1). However, after false discovery rate correction, only (1R,3R,4S)-2,2,4-trimethylbicyclo[2.2.1]heptan-3-ol was found to differ between eggs carrying embryos of either sex (Table 1). In general, eggs with a female embryo were found to release larger amounts of volatiles (Fig 2). Importantly, the effect size associated to sex in these models was “large” [53] for as many as 8 compounds, while it was “intermediate” for 7 compounds. Hence, the effect of sex on odour composition was at least intermediate for approximately 50% of the 31 compounds (Table 1). The mean effect size (*Z*_*r*_) was -0.3163 (95% confidence interval: -0.4066−-0.2260), implying that across all compounds the effect size of sex was “intermediate” and that females released on average more volatiles than males as shown by the fact that the 95% confidence interval did not encompass 0. For two compounds, hexanal and butanoic acid, the effect of embryo mass on the concentration of volatiles was associated to p < 0.05. However, none of these effects were significant after false discovery rate adjustment. The effect sizes associated to body mass for these compounds were 0.644 and 0.547 respectively.

**Table 1:** Linear mixed models of variation in the concentration of volatile compounds between barn swallow eggs containing embryos of either sex.

Class	Volatile Compound	m	f	χ^2^	Effect	F	df	p	Effect size (r)
*Ketones*	Propan-2-one	12	7	0.03	sex	0.27	1, 16.1	0.789	0.134
					mass^§^	3.31	1, 9	0.100	
	6-methyl-5-heptan-2-one	11	7	0	sex	0.02	1, 16	0.916	0.037
					mass^§^	0.61	1, 15	0.446	
	1-phenylethanone	11	7	0	sex	3.38	1, 16	0.164	-0.438
					mass^§^	0.01	1, 5.6	0.924	
*Hydrocarbons*	Pentadecane	11	7	3.02	sex	5.80	1, 13.3	0.119^$^	-0.533
					mass^§^	0.05	1, 14.7	0.832	
	Tetradecane	11	7	2.12	sex	5.37	1, 12.6	0.119^$^	-0.514
					mass^§^	0.15	1, 14.8	0.705	
*Terpenes*	4,7,7-trimethylbicyclo[2.2.1] heptan-3-one	12	7	0.5	sex	0.02	1, 15.2	0.916	-0.038
					mass^§^	2.32	1, 15.7	0.148	
	(4,7,7-trimethyl-3-bicyclo [2.2.1]heptanyl) acetate	12	5	**8.91**^$^	sex	1.56	1, 11.2	0.369	-0.154
					mass^§^	0.12	1, 12.1	0.735	
	(1R,3R,4S)-2,2,4-trimethyl bicyclo [2.2.1]heptan-3-ol	11	7	**9.68**^$^	sex	**18.12**	**1, 12.4**	**0.032**^$^	-0.542
					mass^§^	0.07	1, 13.5	0.792	
*Ammides*	Acetamide	12	7	1.08	sex	3.88	1, 17	0.156	-0.455
					mass^§^	0.38	1, 16	0.546	
	Formamide	10	7	0	sex	8.78	1, 11.6	0.119^$^	-0.627
					mass^§^	0.04	1, 11.7	0.845	
*Alcohols*	Methanethiol	12	7	4.35^$^	sex	0.11	1, 13.9	0.895	-0.080
					mass^§^	0.45	1, 16	0.511	
	Hexan-3-ol	12	7	**12.86**^$^	sex	0.41	1, 13.2	0.721	0.063
					mass^§^	0.01	1, 14.1	0.920	
	3-methyl-1-butanol	11	7	3.42	sex	0.02	1, 12.7	0.916	-0.033
					mass^§^	1.42	1, 14.8	0.253	
	Butane-1,3-diol	12	7	0.06	sex	0.12	1, 15.2	0.895	-0.088
					mass^§^	0.28	1, 10.7	0.610	
	6-ethyl-3-octanol	12	6	3.39	sex	3.65	1, 12.5	0.164	-0.443
					mass^§^	4.49	1, 13.4	0.053	
	5-methyl-3-hexanol	12	7	**14.31**^$^	sex	5.67	1, 13.3	0.119^$^	-0.250
					mass^§^	0.00	1, 14.3	0.989	
	Phenol	11	7	5.05^$^	sex	2.18	1, 12.6	0.283	-0.320
					mass^§^	1.24	1, 15	0.283	
*Aldehydes*	Hexanal	11	6	2.65	sex	1.81	1, 10.9	0.336	0.353
					mass	8.99	1, 13.5	0.010	
	Heptanal	11	6	4.76^$^	sex	1.46	1, 11.6	0.371	-0.274
					mass^§^	0.15	1, 13.9	0.707	
	Octanal	11	7	0.79	sex	0.01	1, 13.7	0.916	-0.028
					mass^§^	1.49	1, 14.5	0.242	
	Nonanal	10	7	2.99	sex	4.55	1, 11.5	0.150	-0.468
					mass^§^	2.38	1, 13.6	0.146	
	Decanal	11	7	**12.12**^$^	sex	6.13	1, 12.2	0.119^$^	-0.267
					mass^§^	1.29	1, 13.8	0.275	
*Free Fatty Acids*	2-methylpropanoic acid	12	7	1.35	sex	0.03	1, 14.3	0.916	0.042
					mass^§^	0.11	1, 14.7	0.747	
	Acetic acid	12	7	0.01	sex	0.86	1, 15.1	0.521	-0.233
					mass^§^	0.18	1, 8.3	0.681	
	Formic acid	9	7	2.33	sex	8.97	1, 10.2	0.119^$^	-0.610
					mass^§^	1.38	1, 10.4	0.267	
	2-methylbutanoic acid	10	7	3.92^$^	sex	5.77	1, 11.4	0.119^$^	-0.421
					mass^§^	1.23	1, 11.9	0.289	
	2,2-dimethylpropanoic acid	12	7	**10.96**^$^	sex	4.30	1, 13.4	0.150	-0.289
					mass^§^	0.48	1, 14.6	0.498	
	Butanoic acid	12	7	0	sex	3.16	1, 16	0.172	-0.430
					mass	5.97	1, 16	0.027	
	5-(dithiolan-3-yl) pentanoic acid	12	7	4.03^$^	sex	7.59	1, 13.9	0.119^$^	-0.565
					mass^§^	0.26	1, 16	0.616	
	2-ethylhexanoic acid	12	7	3.22	sex	5.99	1, 14.2	0.119^$^	-0.533
					mass^§^	0.07	1, 15.6	0.798	
	Octanoic acid	12	7	0	sex	3.57	1, 17	0.164	-0.440
					mass^§^	0.07	1, 16	0.793	

m: number of males; f: number of females.

Sample size varies because of the exclusion of outliers.

The effect of the nest identity, included in all the models as a random factor, was computed by using likelihood ratio tests (χ^2^ values).

Boldface indicates tests associated with p < 0.05 after false discovery rate correction.

^$^ indicates test that were significant (p < 0.05) before false discovery rate correction.

^§^ indicates that embryo mass was removed from the model because its effect was associated with p ≥ 0.05.

A positive sign of effect size means that the volatile compound concentration is higher in male as compared to female embryos.

For 11 out of the 31 compounds analysed, the effect of nest was associated with p < 0.05 at likely ratio test. Six of these tests were still significant after false discovery rate adjustment, implying significant among-nests variation in emission of volatiles from the eggs.

The comparisons between eggshell halves where we tested for accumulation of environmental compounds showed that in no case the concentration of the focal compounds in the eggshell halves that were placed in a nest in the cowsheds where barn swallows nest was significantly larger than that recorded for eggshells that were kept in the lab (p ≥ 0.05 in all paired t-tests) (Table 2). The *unsigned* effect size were “large” in 11 cases, “intermediate” in 12 cases and “small” in the remaining 6 cases. It should be noted, however, that the sign of the difference in volatile concentration between the two conditions was inconsistent across compounds. In addition, the concentration of volatiles was found to be larger, on average, in the lab-stored as compared to cowshed-stored eggshells (mean effect size (*Z*_*r*_): 0.2779; 95% confidence interval: 0.0967−0.4590). Moreover, the compounds for which a significant effect of nest on volatiles concentration was detected were invariably found to be more concentrated in the lab-stored compared to the cowshed-stored eggshells. These findings suggest that environment *per se* did not markedly affect the analyses of among-nests variation in odour. In addition, we emphasize that environmental effect on odour, if any, could not affect the outcome of the analyses of the effect of sex, because nest was included as random effect in the models, and all clutches contained both male and female embryos.

**Table 2:** Paired t-test of the difference in the concentration of volatile compounds between eggshell halves placed in nests in the cowshed and eggshells kept in the lab.

Class	Volatile Compound	Mean lab	Mean nest	t	df	p	Effect size (r)
*Ketones*	Propan-2-one	65.57	69.65	-0.96	3	0.675	-0.485
	6-methyl-5-heptan-2-one	1.16	0.97	0.44	4	0.812	0.215
	1-phenylethanone	0.09	0.11	-0.71	4	0.692	-0.335
*Hydrocarbons*	Pentadecane	0.03	0.01	0.77	3	0.675	0.406
	Tetradecane	0.19	0.15	1.46	4	0.675	0.590
*Terpenes*	4,7,7-trimethylbicyclo[2.2.1]heptan-3-one	0.13	0.28	-1.10	4	0.675	-0.482
	(4,7,7-trimethyl-3-bicyclo [2.2.1]heptanyl) acetate	0.71	0.22	1.20	4	0.675	0.514
	(1R,3R,4S)-2,2,4-trimethylbicyclo [2.2.1]heptan-3-ol	3.02	1.77	1.01	4	0.675	0.451
*Ammides*	Acetamide	0.42	0.41	0.14	3	0.931	0.081
	Formamide	0.26	0.17	1.46	3	0.675	0.645
*Alcohols*	Methanethiol	0.04	0.09	-0.55	3	0.774	-0.303
	Hexan-3-ol	0.52	0.13	1.07	3	0.675	0.526
	3-methyl-1-butanol	0.00	0.02	-1.00	3	0.675	-0.500
	Butane-1,3-diol	0.31	0.17	1.59	3	0.675	0.676
	6-ethyl-3-octanol	0.89	0.87	0.03	3	0.971	0.017
	5-methyl-3-hexanol	1.59	0.69	1.41	4	0.675	0.576
	Phenol	1.11	0.15	1.75	3	0.675	0.711
*Aldehydes*	Hexanal	0.51	0.28	0.96	3	0.675	0.485
	Heptanal	0.20	0.52	-1.17	4	0.675	-0.505
	Octanal	0.14	0.13	0.26	3	0.931	0.148
	Nonanal	2.02	2.08	-0.13	4	0.931	-0.065
	Decanal	0.11	0.08	0.73	3	0.692	0.388
*Free Fatty Acids*	2-methylpropanoic acid	1.06	1.00	0.18	4	0.931	0.090
	Acetic acid	3.48	2.00	1.73	4	0.675	0.654
	Formic acid	0.30	0.15	1.60	3	0.675	0.679
	2-methylbutanoic acid	0.32	0.17	0.95	3	0.675	0.481
	2,2-dimethylpropanoic acid	0.76	0.52	0.77	4	0.692	0.359
	Butanoic acid	0.02	0.01	0.70	3	0.692	0.375
	5-(dithiolan-3-yl)pentanoic acid	0.14	0.11	0.75	3	0.5692	0.397
	2-ethylhexanoic acid	0.16	0.04	1.03	3	0.675	0.511
	Octanoic acid	0.02	0.02	1.74	3	0.675	0.709

Degrees of freedom vary because of the exclusion of outliers.

A positive sign of effect size means that the volatile compound concentration was higher in the eggshell kept in the lab as compared to the eggshell placed in the cowshed.

## Discussion

In the present study we showed, for the first time in any bird species in the wild, that odour composition varies among sibling eggs depending on sex of the embryo, as well as among clutches laid by different females. In fact, although only one out of the 31 volatile compounds that we scrutinized was found to be significantly more concentrated in eggs carrying a female after false discovery rate correction for multiple comparisons, a large proportion of the concentrations were found to be larger for female, the tests of sex differences were associated to “intermediate” or “large” effect sizes [50] for ca. 50% of the compounds, and the mean effect size significantly deviated from 0. This suggests that lack of statistical significance was due to Type II statistical errors arising because, for ethical reasons, sample size was small, thereby reducing the statistical power of the tests, and because of the false discovery rate adjustment of p-values.

The difference in odour composition among eggs containing embryos of either sex can be explained by several non-mutually exclusive mechanisms. As suggested by the only study that characterized the odour composition of bird eggs [26], the main source of sex-related differences in egg odour could be the differential deposition of maternal resources to the eggs of either sex [54–56]. This mechanism may apply to barn swallows, where the deposition of maternal antibodies in the eggs has been found to be associated with the sex of the embryo [54]. However, no sex-dependent variation has been found in the concentration of carotenoids [54] or androgens [57] in barn swallow eggs. An alternative mechanism may consist of general differences in physiology between males and females that cause, as a side effect, sex-related variation in odour composition. Related to general sex-dependent variation in physiology, differences in odour between males and females may also arise as a consequence of differences in growth rate of embryos of either sex, which has been shown to occur in several eutherian mammals [58–60] and, recently, also in birds [61]. Considerable sex differences in the level of gene expression have been detected in birds from the beginning of embryo development [62–64], even before the development of the uropygial gland [65], which is a major source of volatiles, and the differentiation of the gonads that, in a small passerine bird (the zebra finch, *Taeniopygia guttata*) with an incubation period comparable to that of the barn swallow, starts at 6.5–7 days of incubation [61]. Male zebra finches, for example, start to grow at a higher rate than females already 36 hours after the start of incubation, due to an overexpression of genes such as the growth hormone receptor gene [61], implied in anabolic processes like lipid degradation, protein synthesis and muscle mass gaining [66], and the FBP1 gene, which is responsible for the production of glucose or glycogen from non-carbohydrates [67]. It should be noticed that, upon dissection, total egg mass was found to be larger for eggs carrying a female. However, embryo mass (excluding yolk) showed a marginally non-significant trend in the opposite direction, with males being heavier than females. This suggests that egg mass reduction during incubation, which is commonly observed in birds, was larger in eggs carrying a male embryo because male embryos have faster development compared to females, consistently with previous observations on the zebra finch. In the context of the present study, this finding suggests that, under the assumption that the volatile compounds that we measured reflect embryonic production, the result of larger production of volatile compounds by female eggs may be conservative because female embryos were smaller than males, on average.

Sex-differences in odour is a prerequisite condition for parental identification of embryo sex via olfactory cues. Whether parents do in fact use such cues to discriminate between sexes already before hatching, thereby modulating their behaviour, is matter of speculation. Indeed, egg odour has been shown to potentially convey a broad spectrum of information, including embryo growth rate and health [68]. Parents have also been shown to accrue information on intra- and inter-specific brood parasitism via odours, suggesting that release of kin-dependent or species-specific odours is a by-product of within- and among-species genetic differences that parents exploit in order to reduce the costs of brood parasitism [14]. Here we showed that, consistent with previous findings [26], parents can also acquire information on sex composition of their clutch in the pre-hatching period. Because in European barn swallow only females incubate, they appear to have more opportunities to exploit any information on odour and modulate their parental behaviour during the pre-hatching period in terms of, for example, nest defence from predators and from egg ejection by other males [43,44], accordingly. However, males also spend considerable amount of time at the nest, for example during the night (personal observation), and may therefore also have the opportunity to acquire and use information on the embryo sex composition of their brood. Incubation behaviour has been demonstrated to have a strong impact on parent reproductive success and future fitness [69,70]. During the incubation period parents have to balance the allocation of resources to self-maintenance against time and energy requirements of incubation [71,72]. Several recent studies have demonstrated diverse effects of incubation on post-natal offspring phenotype. For example, DuRant and co-workers [73] experimentally manipulated the incubation temperature of wood ducks (*Aix sponsa*) and found that small differences in incubation temperature affect nestling survival, growth rate, body condition and stress-induced corticosterone levels. In the blue tit (*Cyanistes caeruleus*), it has also been demonstrated that incubation temperature affects, besides nestling morphology, nestling metabolic rate [74]. Because embryos of either sex may inherently differ in pre-natal growth trajectories ([61] and present study) and might also differ in susceptibility to parental incubation behaviour, pre-natal identification of embryo sex may allow parents to optimize incubation according to clutch sex composition and/or sex of individual embryos.

There are good reasons to speculate that parents should use sex-specific chemical information before hatching in order to tune their parental behaviour. Sex allocation theory predicts differential parental investment in relation to the reproductive value of the offspring [30,75,76]. The expected fitness returns from male and female offspring can vary according to a number of factors that range from paternal/maternal genetic and/or phenotypic quality [77–80], to ecological conditions [81–83] and population sex ratio [84,85]. In the barn swallow, previous studies have shown that differential parental allocation occurs depending on offspring sex [86,87]. It has also been demonstrated that nestling vocalizations [37] and gape colouration [38] varies between the sexes. Recent studies have also revealed significant differences in ventral colour between nestling of either sex [88,89] and differential variation in parental care allocation in relation to offspring plumage colouration among males as compared to female nestlings [90]. The existence of sex-related variation in offspring post-hatching phenotype and of differential allocation to either sex by parents suggests that sex-dependent egg odour might pave the way to pre-hatching differential allocation of care by parents. Admittedly, whether parents do in fact use this information to adaptively modulate their behaviour remains to be elucidated, because neither the present study nor the study by Webster et al. [26], that was carried out in the lab on artificially incubated eggs, have tested for an effect of sex-dependent odours on parental behaviour. We also suspect that any such experiment will be technically hardly feasible, owing to the difficulty of manipulating the concentration of volatile compounds within the natural range of variation in the wild. An alternative possibility could be to experimentally produce unisexual clutches by swapping eggs between nests, though this approach would likely require *a posteriori* identification of unisexual broods because non-invasively sexing the embryo of developing eggs at present is not technically feasible, particularly at early developmental stages.

In designing this study, we speculated that much variation in egg odour could be generated by variation in nesting micro-habitat, an hypothesis that has not been tested in the past because previous studies kept the eggs under artificial incubation conditions. The effects of environmental odours are particularly likely to occur for barn swallows because they mostly breed in cowsheds often with limited air circulation and presence of cows and manure. Comparison between eggshells that were either kept in plastic bags or left in an unoccupied nest and exposed to cowshed odours did not reveal any significant difference. Effect sizes of the differences between eggshells kept in the lab or in a cowshed were large but did not reveal a consistent tendency towards cowshed eggshells releasing more volatiles. In addition, there was no evidence that the compounds that differed the most among nests were those that were also more abundantly released by cowshed as compared to lab-stored eggshells. These results suggest that microhabitat was not a major confound, and that among-nests variation reflects the genuine phenotypic and/or genetic influences of parentage on egg odour. Genetic variation in odour profiles at family level has been consistently demonstrated in vertebrates and has been attributed in some studies to polymorphism at the major histocompatibility complex (MHC) [91,92]. The observed consistency in odour within nest may be part of mechanisms of kin recognition which are established early in life. Kin recognition, in turn, may function to reduce the risk of inbreeding. However, we speculate that kin discrimination is unlikely to play a major role in mate choice in our model species because natal dispersal is high and female biased [44,93], whereas breeding dispersal is very low, implying that both mating between siblings and between parents and offspring is unlikely. Indeed, siblings of different sex are very seldom found breeding in the same colony and the few, mostly male, offspring that are recruited in their natal colony tend to be reproductively isolated from their parents because older individuals breed earlier than 1-year old recruits [43,44].

In conclusion, the present study is the first to show sex-related odour differences in the eggs in any vertebrate species in the wild. While this observation implies that parents have a cue to discriminate between eggs carrying embryos of either sex, whether parents do indeed use it and modulate their behaviour accordingly remains to be elucidated. Finally, variation in odour composition among nests, that apparently did not depend on nesting micro-habitat, indicates that the prerequisite conditions for kin recognition based on olfactory cues exist in our model species.

## Supporting Information

S1 Dataset The file summarizes all the relevant data that have been used in the statistical analyses. (XLSX)

## References

[1] Wenzel BM. Chemoreception In: Farner DS, King JR editors. Avian Biology. Academic Press, New York & London; 1973 pp. 389–415.

[2] Otte D. Effects and functions in the evolution of signaling systems. Annu Rev Ecol Syst. 1974; 5: 385–417.

[3] Hagelin JC, Jones IL. Bird odors and other chemical substances: a defense mechanism or overlooked mode of intraspecific communication? Auk; 2007; 10.1642/0004-8038(2007)124[741:boaocs]2.0.co;2

[4] Bang BG. Anatomical evidence for olfactory function in some species of birds. Nature. 1960; 188: 547–549. 13686568 10.1038/188547a0

[5] Wenzel BM. The olfactory prowess of the kiwi. Nature. 1968; 220: 1133–1134. 10.1038/2201133a0 5723611

[6] Caro SP, Balthazart J. Pheromones in birds: myth or reality? J Comp Physiol A. 2010; 196: 751–766. 10.1007/s00359-010-0534-4 PMC3522863 20490809

[7] Bang BG, Wenzel BM. Nasal cavity and olfactory system In: King AS, McLelland J, editors. Form and function in birds. New York Academic Press 1985 pp. 195–225.

[8] Steiger SS, Fidler AE, Kempenaers B. Evidence for increased olfactory receptor gene repertoire size in two nocturnal bird species with well-developed olfactory ability. BMC Evol Biol. 2009; 9: 117 10.1186/1471-2148-9-117 19467156 PMC2701422

[9] Stager KE. The role of olfaction in food location by the turkey vulture (*Cathartes aura*). Los Angeles County Mus Contrib Sci. 1964; 81: 1–63.

[10] Grubb TC. Smell and foraging in shearwaters and petrels. Nature. 1972; 237: 404–405. 10.1038/237404a0

[11] Bang BG, Cobb S. The size of the olfactory bulb in 108 species of birds. Auk. 1968; 85: 55–61. 10.2307/4083624

[12] Bonadonna F, Sanz-Aguilar A. Kin recognition and inbreeding avoidance in wild birds: the first evidence for individual kin-related odour recognition. Anim Behav. 2012; 84: 509–513. 10.1016/j.anbehav.2012.06.014

[13] Krause ET, Brummel C, Kohlwey S, Baier MC, Müller C, Bonadonna F, et al Differences in olfactory species recognition in the females of two Australian songbird species. Behav Ecol Sociobiol. 2014; 68: 1819–1827. 10.1007/s00265-014-1791-y

[14] Soler JJ, Pérez‐Contreras T, De Neve L, Macías‐Sánchez E, Møller AP, Soler M. Recognizing odd smells and ejection of brood parasitic eggs. An experimental test in magpies of a novel defensive trait against brood parasitism. J Evol Biol. 2014; 27: 1265–1270. 10.1111/jeb.12377 24725170

[15] Gagliardo A. Forty years of olfactory navigation in birds. J Exp Biol. 2013; 216: 2165–2171. 10.1242/jeb.070250 23720797

[16] Nevitt GA. Sensory ecology on the high seas: the odor world of the procellariiform seabirds. J Exp Biol. 2008; 211: 1706–1713. 10.1242/jeb.015412 18490385

[17] Bonadonna F, Cunningham GB, Jouventin P, Hesters F, Nevitt GA. Evidence for nest-odour recognition in two species of diving petrel. J Exp Biol. 2003; 206: 3719–3722. 10.1242/jeb.00610 12966063

[18] Amo L, Avilés JM, Parejo D, Peña A, Rodríguez J, Tomás G. Sex recognition by odour and variation in the uropygial gland secretion in starlings. J Anim Ecol. 2012; 81: 605–613. 10.1111/j.1365-2656.2011.01940.x 22220811

[19] Jacob J. Hydrocarbon and multibranched ester waxes from the uropygial gland secretion of grebes (Podicipediformes). J Lipid Res. 1978; 19: 148–153. 344824

[20] Whittaker DJ, Richmond KM, Miller AK, Kiley R, Burns CB, Atwell JW, et al Intraspecific preen oil odor preferences in dark-eyed juncos (*Junco hyemalis*). Behav Ecol. 2011; arr122. 10.1093/beheco/arr122

[21] Balthazart J, Schoffeniels E. Pheromones are involved in the control of sexual behaviour in birds. Naturwissenschaften. 1979; 66: 55–56. 10.1007/BF00369365 370614

[22] Hirao A, Aoyama M, Sugita S. The role of uropygial gland on sexual behavior in domestic chicken *Gallus gallus domesticus*. Behav Processes. 2009; 80: 115–120. 10.1016/j.beproc.2008.10.006 19013507

[23] Cohen J. Olfaction and parental behavior in ring dove. Biochem Syst Ecol. 1981; 9: 351–354. 10.1016/0305-1978(81)90022-3

[24] Parejo D, Amo L, Rodríguez J, Avilés JM. Rollers smell the fear of nestlings. Biol Lett. 2012; 8: 502–504. 10.1098/rsbl.2012.0124 22399785 PMC3391467

[25] Golüke S, Dörrenberg S, Krause ET, Caspers BA. Female Zebra Finches smell their eggs. PLoS One. 2016; 11: e0155513 10.1371/journal.pone.0155513 27192061 PMC4871452

[26] Webster B, Hayes W, Pike TW. Avian egg odour encodes information on embryo sex, fertility and development. PLoS One. 2015; 10: e0116345 10.1371/journal.pone.0116345 25629413 PMC4309571

[27] Fisher RA. The genetical theory of natural selection Oxford University Press; 1930.

[28] Trivers R, Willard DE. Natural selection of parental ability to vary the sex ratio of offspring. Science. 1973; 179: 90–92. 10.1126/science.179.4068.90 4682135

[29] Charnov EL. The theory of sex allocation Princeton University Press; 1982 7144766

[30] West SA. Sex Allocation. Princeton: Princeton University Press; 2009

[31] Strehl CE, White J. Effects of superabundant food on breeding success and behavior of the red-winged blackbird. Oecologia. 1986; 70: 178–186. 10.1007/BF00379237 28311655

[32] Dijkstra C, Daan S, Pen I. Fledgling sex ratios in relation to brood size in size-dimorphic altricial birds. Behav Ecol. 1998; 9: 287–296. 10.1093/beheco/9.3.287

[33] Mainwaring MC, Dickens M, Hartley IR. Sexual dimorphism and offspring growth: smaller female Blue Tit nestlings develop relatively larger gapes. J Ornithol. 2012; 153: 1011–1016. 10.1007/s10336-012-0828-0

[34] Roulin A, Richner H, Ducrest AL. Genetic, environmental, and condition-dependent effects on female and male ornamentation in the barn owl *Tyto alba*. Evolution. 1998; 52: 1451–1460. 10.2307/2411314 28565392

[35] Johnsen A, Delhey K, Andersson S, Kempenaers B. Plumage colour in nestling blue tits: sexual dichromatism, condition dependence and genetic effects. Proc R Soc Lond B. 2003; 270: 1263–1270. 10.1098/rspb.2003.2375 PMC1691364 12816639

[36] Monk DS, Koenig WD, Koenig WR. Individual, brood, and sex variation in begging calls of western bluebirds. Wilson Bull. 1997; 109: 328–332.

[37] Saino N, Galeotti P, Sacchi R, Boncoraglio G, Martinelli R, Møller AP. Sex differences in begging vocalizations of nestling barn swallows, *Hirundo rustica*. Anim Behav. 2003; 66: 1003–1010. 10.1006/anbe.2003.2295

[38] Saino N, De Ayala RM, Boncoraglio G, Martinelli R. Sex difference in mouth coloration and begging calls of barn swallow nestlings. Anim Behav. 2008; 75: 1375–1382. 10.1016/j.anbehav.2007.09.011

[39] Paganelli CV. The physics of gas exchange across the avian eggshell. Am Zool. 1980; 20: 329–338.

[40] Vleck CM, Bucher TL. Energy metabolism, gas exchange, and ventilation In: Stark JM, Ricklefs RE editors. Avian growth and development. Evolution within the precocial-altricial spectrum. Oxford University Press; 1998 pp. 89–116

[41] Spurr EB Developing a long-life toxic bait and lures for mustelids. Science for Conservation. 1999; 127A: 1–24.

[42] Cramp S. The Complete Birds of the Western Palearctic on CD-ROM. Oxford University Press, Oxford; 1998.

[43] Møller AP. Sexual Selection and the Barn Swallow. Oxford University Press; 1994.

[44] Turner A. The Barn Swallow. T & AD Poyser, London; 2006.

[45] Saino N, Martinelli R, Romano M. Ecological and phenological covariates of offspring sex ratio in barn swallows. Evol Ecol. 2008; 22: 659–674. 10.1007/s10682-007-9189-1

[46] Manzo A, Panseri S, Vagge I, Giorgi A. Volatile fingerprint of Italian populations of orchids using solid phase microextraction and gas chromatography coupled with mass spectrometry. Molecules. 2014; 19: 7913–7936. 10.3390/molecules19067913 24962394 PMC6271603

[47] Panseri S, Soncin S, Chiesa LM, Biondi PA. A headspace solid-phase microextraction gas-chromatographic mass-spectrometric method (HS-SPMEGC/MS) to quantify hexanal in butter during storage as marker of lipid oxidation. Food Chem. 2011; 127: 886–889. 10.1016/j.foodchem.2010.12.150 23140750

[48] Benjamini Y, Hochberg Y. Controlling the false discovery rate: a practical and powerful approach to multiple testing. J R Statis Soc B. 1995; 57: 289–300

[49] Garamszegi LZ. Comparing effect sizes across variables: generalization without the need for Bonferroni correction. Behav Ecol. 2006; 17: 682–687. 10.1093/beheco/ark005

[50] Nakagawa S, Cuthill IC. Effect size, confidence interval and statistical significance: a practical guide for biologists. Biol Rev. 2007; 82: 591–605. 10.1111/j.1469-185X.2007.00027.x 17944619

[51] Rosenberg MS, Adams DC, Gurevitch J. MetaWin: statistical software for meta-analysis Sunderland, Massachusetts: Sinauer Associates, Inc; 2000.

[52] Viechtbauer W. Conducting meta-analyses in R with the metafor Package. J Stat Softw. 2010; 36: 1–48. 10.18637/jss.v036.i03

[53] Cohen J. Statistical power analysis for the behavioural sciences NY: Academy Press: New York, 1988.

[54] Saino N, Romano M, Ferrari RP, Martinelli R, Møller AP. Maternal antibodies but not carotenoids in barn swallow eggs covary with embryo sex. J Evolution Biol. 2003; 16: 516–522. 10.1046/j.1420-9101.2003.00534.x 14635852

[55] Gilbert L, Rutstein AN, Hazon N, Graves JA. Sex-biased investment in yolk androgens depends on female quality and laying order in zebra finches (*Taeniopygia guttata*). Naturwissenschaften. 2005; 92: 178–181. 10.1007/s00114-004-0603-z 15668780

[56] Badyaev AV, Seaman DA, Navara KJ, Hill GE, Mendonca MT. Evolution of sex-biased maternal effects in birds: III. Adjustment of ovulation order can enable sex-specific allocation of hormones, carotenoids, and vitamins. J Evol Biol. 2006; 19: 1044–1057. 10.1111/j.1420-9101.2006.01106.x 16780506

[57] Gil D, Ninni P, Lacroix A, De Lope F, Tirard C, Marzal A, et al Yolk androgens in the barn swallow (*Hirundo rustica*): a test of some adaptive hypotheses. J Evol Biol. 2006; 19: 123–131. 10.1111/j.1420-9101.2005.00981.x 16405584

[58] Bernardi ML, Delouis C. Sex-related differences in the developmental rate of in-vitro matured/in-vitro fertilized ovine embryos. Hum Reprod. 1996; 11: 621–626. 10.1093/humrep/11.3.621 8671280

[59] Peippo J, Bredbacka P. Sex-related growth rate differences in mouse preimplantation embryos in vivo and in vitro. Mol Reprod Dev. 1995; 40: 56–61. 10.1002/mrd.1080400108 7702870

[60] Ménézo YJR, Chouteau J, Torelló MJ, Girard A, Veiga A. Birth weight and sex ratio after transfer at the blastocyst stage in humans. Fertil Steril. 1999; 72: 221–224. 10.1016/s0015-0282(99)00256-3 10438983

[61] Tagirov M, Rutkowska J. Sexual dimorphism in the early embryogenesis in zebra finches. PLoS One. 2014; 9: e114625 10.1371/journal.pone.0114625 25493645 PMC4262425

[62] Mank JE, Ellegren H. All dosage compensation is local: gene-by-gene regulation of sex-biased expression on the chicken Z chromosome. Heredity. 2009; 102: 312–320. 10.1038/hdy.2008.116 18985062

[63] Zhang SO, Mathur S, Hattem G, Tassy O, Pourquié O. Sex-dimorphic gene expression and ineffective dosage compensation of Z-linked genes in gastrulating chicken embryos. BMC Genomics. 2010; 11: 13 10.1186/1471-2164-11-13 20055996 PMC2821371

[64] Zhao D, McBride D, Nandi S, McQueen HA, McGrew MJ, Hocking PM, et al Somatic sex identity is cell autonomous in the chicken. Nature. 2010; 464: 237–242. 10.1038/nature08852 20220842 PMC3925877

[65] Jacob J, Ziswiler V The uropygial gland In: Farner DS, King JR editors. Avian Biology. Academic Press, New York & London; 1982 pp. 199–324.

[66] Van Kerkhof P, Putters J, Strous GJ. The ubiquitin ligase SCF(betaTrCP) regulates the degradation of the growth hormone receptor. J Biol Chem. 2007; 282: 20475–20483. 10.1074/jbc.M702610200 17500058

[67] Tillmann H, Bernhard D, Eschrich K. Fructose-1,6-bisphosphatase genes in animals. Gene. 2002; 291: 57–66. 10.1016/S0378-1119(02)00627-3 12095679

[68] Surai PF. Natural antioxidants in avian nutrition and reproduction Nottingham University Press; 2002.

[69] Williams JB Energetics of avian incubation In: Carey C, editor. Avian Energetics and Nutritional Ecology. New York: Chapman and Hall; 1996 pp. 375–416.

[70] Tinbergen JM, Williams JB. Energetics of incubation In: Deeming DC, editor. Avian Incubation: Behaviour, Environment, and Evolution. Oxford: Oxford University Press; 2002 pp. 299–313.

[71] Carey C. The ecology of avian incubation. BioScience. 1980; 30: 819–824. 10.2307/1308374

[72] Deeming DC. Behavior patterns during incubation In: Deeming DC, editor. Avian Incubation: behaviour, environment, and evolution. Oxford: Oxford University Press; 2002 pp. 63–87.

[73] DuRant SE, Hepp GR, Moore IT, Hopkin BC, Hopkins WA. Slight differences in incubation temperature affect early growth and stress endocrinology of wood duck (*Aix sponsa*) ducklings. J Exp Biol. 2010; 213: 45–51. 10.1242/jeb.034488 20008361

[74] Nord A, Nilsson JÅ. Incubation temperature affects growth and energy metabolism in blue tit nestlings. Am Nat. 2011; 178: 639–651. 10.1086/662172 22030733

[75] Frank SA. Sex allocation theory for birds and mammals. Annu Rev Ecol Syst. 1990; 21: 13–55.

[76] Komdeur J. Sex allocation In: Royle N, Smiseth, Koelliker M editors. The Evolution of Parental Care. Oxford University Press, Oxford; 2012 pp. 171–181

[77] Nager RG, Monaghan P, Griffiths R, Houston DC, Dawson R. Experimental demonstration that offspring sex ratio varies with maternal condition. Proc Natl Acad Sci USA. 1999; 96: 570–573. 10.1073/pnas.96.2.570 9892674 PMC15177

[78] Tschirren B, Postma E, Rutstein AN, Griffith SC. When mothers make sons sexy: maternal effects contribute to the increased sexual attractiveness of extra-pair offspring. Proc R Soc Lond B. 2012; 279: 1233–1240. 10.1098/rspb.2011.1543 PMC3267142 21957136

[79] Booksmythe I, Mautz B, Davis J, Nakagawa S, Jennions MD. Facultative adjustment of the offspring sex ratio and male attractiveness: a systematic review and meta-analysis. Biol Rev. 2015 10.1111/brv.12220 26405787

[80] Romano A, Romano M, Caprioli M, Costanzo A, Parolini M, Rubolini D, et al Sex allocation according to multiple sexually dimorphic traits of both parents in the barn swallow (*Hirundo rustica*). J Evol Biol. 2015; 28: 1234–1247. 10.1111/jeb.12650 25913917

[81] Appleby BM., Petty SJ, Blakey JK, Rainey P, Macdonald DW. Does variation of sex ratio enhance reproductive success of offspring in tawny owls (*Strix aluco*)? Proc R Soc Lond B. 1997; 264: 1111–1116. 10.1098/rspb.1997.0153

[82] Komdeur J, Daan S, Tinbergern J, Mateman AC. Extreme adaptive modification in sex ratio of the Sychelles warbler’s eggs. Nature. 1997; 385: 522–526. 10.1038/385522a0

[83] Romano A, Ambrosini R, Caprioli M, Bonisoli-Alquati A, Saino, N. Secondary sex ratio covaries with demographic trends and ecological conditions in the barn swallow. Evol Ecol. 2012; 26: 1041–1053. 10.1007/s10682-011-9543-1

[84] Cordero PJ, Vinuela J, Aparicio JM, Veiga JP. Seasonal variation in sex ratio and sexual egg dimorphism favouring daughters in first clutches of the spotless starling. J Evol Biol. 2001; 14: 829–834. 10.1046/j.1420-9101.2001.00320.x

[85] Uller T. Sex-specific sibling interactions and offspring fitness in vertebrates: patterns and implications for maternal sex ratios. Biol Rev. 2006; 81: 207–217. 10.1017/S1464793105006962 16677432

[86] Boncoraglio G, Martinelli R, Saino N. Sex-related asymmetry in competitive ability of sexually monomorphic barn swallow nestlings. Behav Ecol Sociobiol. 2008; 62: 729–738. 10.1007/s00265-007-0498-8

[87] Bonisoli-Alquati A, Boncoraglio G, Caprioli M, Saino N. Birth order, individual sex and sex of competitors determine the outcome of conflict among siblings over parental care. Proc R Soc Lond B. 2011; 278: 1273–1279. 10.1098/rspb.2010.1741 PMC3049075 20943688

[88] Hubbard JK, Jenkins BR, Safran RJ. Quantitative genetics of plumage color: lifetime effects of early nest environment on a colorful sexual signal. Ecol Evol. 2015; 5: 3436–3449. 10.1002/ece3.1602 26380676 PMC4569038

[89] Costanzo A, Parolini M, Bazzi G, Khoriauli L, Santagostino M, Possenti CD et al Brood size, telomere length and parent-offspring color signaling in barn swallows. Behav Ecol. 2016 10.1093/beheco/arw147

[90] Romano A, Bazzi G; Caprioli M, Corti M, Costanzo A, Rubolini D et al Nestling sex and plumage color predict food allocation by barn swallow parents. Behav Ecol. 2016; 27:1198–1205. 10.1093/beheco/arw040

[91] Yamazaki K, Beauchamp GK, Curran M, Bard J, Boyse EA. Parent–progeny recognition as a function of MHC odortype identity. Proc Natl Acad Sci USA. 2000; 97: 10500–10502. 10.1073/pnas.180320997 10973487 PMC27053

[92] Jacob S, McClintock MK, Zelano B, Ober C. Paternally inherited HLA alleles are associated with women's choice of male odor. Nature Genet. 2002; 30: 175–179. 10.1038/ng830 11799397

[93] Balbontín J, Møller AP, Hermosell IG, Marzal A, Reviriego M, De Lope F. Geographic patterns of natal dispersal in barn swallows *Hirundo rustica* from Denmark and Spain. Behav Ecol Sociobiol. 2009; 63: 1197–1205. 10.1007/s00265-009-0752-3

